# Evaluation of a peer-led research best practices training for community health workers and promotoras

**DOI:** 10.1017/cts.2024.593

**Published:** 2024-09-16

**Authors:** Susan L. Murphy, Alexandra E. Harper, Gina M. Jay, Vanessa I. Trujillo, Kristen Weeks-Norton, Elias Samuels, Jonathan P. Troost, Brenda Eakin, Gretchen Piatt, Catherine Striley, Analay Perez, Shannen McIntosh, Daphne C. Watkins, Sergio Aguilar-Gaxiola, Linda Cottler

**Affiliations:** 1 Michigan Institute of Clinical and Health Research, University of Michigan, Ann Arbor, MI, USA; 2 Department of Physical Medicine and Rehabilitation, University of Michigan, Ann Arbor, MI, USA; 3 Clinical and Translational Science Center, University of California, Davis, CA, USA; 4 Center for Reducing Health Disparities, University of California, Davis, CA, USA; 5 Department of Learning Health Sciences, Department of Health Behavior and Health Education, School of Public Health, University of Michigan, Ann Arbor, MI, USA; 6 Department of Epidemiology, College of Public Health and Health Professions and College of Medicine, University of Florida, Gainesville, FL, USA; 7 Department of Family Medicine, University of Michigan, Ann Arbor, MI, USA; 8 School of Social Work, University of Michigan, Ann Arbor, MI, USA; 9 Department of Internal Medicine, Center for Reducing Health Disparities and Clinical and Translational Science Center, University of California, Davis, CA, USA

**Keywords:** Community health workers, promotoras, research training, research competency, cultural adaptation

## Abstract

**Introduction:**

Community health workers and promotoras (CHW/Ps) increasingly support research conducted in communities but receive variable or no training. We developed a culturally and linguistically tailored research best practices course for CHW/Ps that can be taken independently or in facilitated groups. The purpose of this study was to evaluate the facilitated training.

**Methods:**

CHW/Ps were recruited from communities and partners affiliated with study sites in Michigan, Florida, and California. They participated in virtual or in-person training facilitated by a peer in English or Spanish and then completed a survey about their abilities (i.e., knowledge and skills for participating in research-related work) and perceptions of the training. Linear regression analyses were used to examine differences in training experience across several factors.

**Results:**

A total of 394 CHW/Ps, mean age 41.6 ± 13.8 years, completed the training and survey (*n* = 275 English; 119 Spanish). Most CHW/Ps were female (80%), and 50% identified as Hispanic, Latino, or Spanish. Over 95% of CHW/Ps rated their abilities as improved after training; 98% agreed the course was relevant to their work and felt the training was useful. Small differences were observed between training sites.

**Discussion:**

Most CHW/Ps rated the training positively and noted improved knowledge and skills for engaging in research-related work. Despite slight site differences, the training was well received, and CHW/Ps appreciated having a facilitator with experience working in community-based settings. This course offers a standard and scalable approach to training the CHW/P workforce. Future studies can examine its uptake and effect on research quality.

## Introduction

Community health workers and promotoras (CHW/Ps), their Spanish-speaking and often bilingual counterparts, are increasingly recognized in translational science for their ability to assist and enhance community-engaged research. They often serve as the bridge between researchers and communities, bringing critical knowledge and insights about communities where research is being planned and conducted [1] and working as trustworthy members of the research team. Their involvement in research teams has improved participant recruitment and retention in studies as well as intervention effectiveness [2]. Even when they are not formal members of research teams, CHW/Ps can help community members to better understand relevant research concepts and empower community members to make informed decisions about participating in the research process. Despite the advantages of engaging CHW/Ps in research, their involvement can result in unintended negative consequences impacting participants. For example, CHW/Ps may not adequately describe randomization, inadequately protect participant privacy or data confidentiality, or potentially coerce community members to participate in studies to increase study enrollment [3]. A high-quality research best practices training, designed specifically for CHW/Ps, could address these and other issues, ultimately increasing scientific rigor and strengthening study teams.

There have been challenges with training CHW/Ps in research. No national research competencies exist for CHW/Ps, and this workforce, while not consistently trained in research best practices, conclusively needs training. Researchers have been unclear on the training needs, how to address them, or what is required for CHW/Ps oversight as part of research teams [2]. In a study of 10 centers engaging CHWs in community-based participatory research, project representatives reported that most CHWs began working without any research training, and while there was a great need for training, it was not prioritized over progressing project goals [2]. Project representatives also reported that they relied on peer mentorship from other CHWs to teach each other necessary knowledge in the field [2]. In addition, the need for relevant cultural and linguistic tailoring of the training is paramount. Manzo and colleagues identified a disconnect between CHW/Ps’ use of research practices and the training available to them to improve their skills. In the English training, which was not the primary language of many of the participant CHW/Ps, they noted a need for more representation of their cultural values and backgrounds that could facilitate participant recruitment and data collection [4]. In other words, CHW/Ps noted a disconnect between themselves and what was being taught, resulting in ineffective training.

Over the past several years, our diverse multisite team worked together to develop a culturally and linguistically tailored research best practices training for CHW/Ps. Our goal was to create an effective training program that is broadly available and accessible to this workforce both in English and Spanish to advance knowledge in and application of research skills. The training was designed to be administered independently or with a facilitator. The independent training is self-paced and only taken virtually. We recently evaluated the independent course and found that nearly all respondents (96–100%) reported improved abilities (i.e., knowledge and skills for participating in research-related work). Similarly, 97% of these CHW/Ps reported that the course was relevant to their work, and 96% rated the training as useful [5]. The facilitated training version, which can be taken virtually or in-person, involves the inclusion of a trained facilitator, also known as a “Champion.” The identified Champion is a CHW/P or an individual who works or volunteers in community-based organizations and provides small group trainings for CHW/Ps. The course content is the same in both the independent and facilitated training versions. The facilitated training offers learners the opportunity to take the course (a) in a small group format with peer CHW/Ps and (b) with a trained facilitator “Champion” who is meant to foster engagement among attendees. Participants can converse and share their lived experiences related to topics covered, which offers additional co-learning opportunities. Otherwise, content and knowledge checks are the same between the online-only independent course and the facilitated version taken online or in-person. The purpose of this study was to evaluate the facilitated training by examining CHW/Ps’ self-rated abilities and perceptions of the training. We were particularly interested in exploring differences in the ratings of the training based on (1) whether it was taken in English or Spanish and (2) the location where training occurred (Michigan, Florida, or California).

## Materials and methods

The course development and content are described in detail elsewhere [5]. Course content (See Table 1) was identified from literature review and conversations with researchers and CHW/Ps [6] and designed to reflect competencies relevant to the CHW/P workforce and was modeled after the Social and Behavioral Research Best Practices Course created for social and behavioral research professionals [7]. A highly participatory approach included involvement from community members from three Clinical and Translational Science Award (CTSA)-funded institutions, University of Michigan (UM), University of Florida (UF), and University of California, Davis (UC Davis), and a variety of partners. The study team included researchers, research staff members, community-based organization leaders, and CHW/Ps from diverse racial, ethnic, and experiential backgrounds who lent critical insight into how to tailor the course content, learning scenarios, and the audio and visual presentation of images and characters throughout to ensure linguistic and cultural appropriateness of the training. The team also collaborated with instructional designers, health literacy experts, and Spanish translation experts to create robust training materials in English and Spanish.

**Table 1.** Overview of the research best practices training for community health workers and promotoras (CHW/Ps)

Module title	Content focus
1. Research Basics*	Fundamentals of the research process, CHW/P roles
2. Recruitment	Engaging community members about participating in research, avoiding coercion
3. Informed Consent	Basics of the informed consent process
4. Privacy & Confidentiality	Strategies to protect participants’ privacy and confidentiality
5. Common Challenges: COVID-19 and Other Public Health Crises*	Identifies common challenges CHW/Ps encounter and how to communicate effectively with community members during crises

CHW/Ps = community health workers and promotoras.

* During the training refinement process, the name for Module 1 was changed to Research and Communities and the name for Module 5 was changed to Community Health and Well-being based on feedback from our partners.

### Community–academic partnership model of training

For the facilitated training, a community–academic partnership approach was used to ensure the development and dissemination of high-quality training. In this partnership, the Champions were paired with a Master Trainer from their respective study site who was a faculty or staff member with experience in research, training, and mentoring. The lead Master Trainer from the UM site trained Master Trainers at the other two study sites. Each site had at least one Master Trainer with extensive prior training and train-the-trainer experience. Train-the-Trainer materials were developed to guide this process.

### Champion selection and training

Each site selected three Champions by collaborating with their community partner networks. Champions were either CHW/Ps or individuals working in community-based organizations affiliated with CHW/Ps who were interested in facilitating the training. No experience in research was necessary if the Champion felt comfortable with the course content. However, experience facilitating group learning among adult learners was a desirable qualification. Depending on the Master Trainer’s availability, Champions participated in either a one-on-one or group orientation at their respective site. Each Champion received all training materials, underwent the same training as their Master Trainer, and was offered ongoing mentorship. Some sites, such as UM, required Champions to lead a mock training with a Master Trainer to ensure the Champion was prepared to lead their training. In other sites, such as UC Davis, the Champions had significant training experience that the requirement was deemed not necessary. Yet, all site Champions were required to complete a self-assessment rating their confidence and knowledge of the material before conducting the training independently.

### Training fidelity

Master Trainers at each site observed, in-person or via recording, the first training day and at least one other training conducted by each Champion at their site. They evaluated training fidelity across 10 characteristics of training performance (e.g., had materials needed to teach the class, engaged with the learners, communicated clearly, fostered group interactions, and provided correct answers to questions). Master Trainers determined if the Champion met the criterion for each characteristic with “no” (rating = 0) or “yes” (rating = 1) and then were required to elaborate on all “no” ratings. Evaluations resulted in a possible score of 0–10, with 10 signifying perfect fidelity. Master Trainers provided remediation to Champions if a training was rated ≤ 7 or at the Champion’s request.

### Participant recruitment

Course participants were CHW/Ps recruited from the community networks in the home states of the study sites. The intended sample size was 405 to be split evenly across the sites. This study was exempted by the University of Michigan Institutional Review Board for all sites.

### Measures

Demographic data collected about the CHW/P participants included age, gender, sex, race, ethnicity, work status, years of experience as a CHW/P, prior research experience (yes/no), and educational attainment. Upon completion of the facilitated-led training, CHW/Ps were asked to rate their abilities (i.e., knowledge and skills) relevant to the training (reflecting the course content in Table 1) and their perceptions related to the usefulness of the training. These measures were used to assess CHW/Ps who took the online training which allowed direct comparison of each delivery method [5]. Both the 10-item self-rated abilities survey and 8-item perceptions survey had excellent to good internal consistency (Cronbach’s alpha = 0.94 and 0.87, respectively). Information was also collected on the course characteristics, such as the modality of training (in-person or virtual), the number of days to complete the full training (1 versus 2 days), and the language in which the course was taken (English or Spanish).

### Data collection

The online registration form included the consent information, and all CHW/Ps confirmed their understanding and interest in participating. To yield as broad a sample as possible, the only identifying information requested from participants in the registration form were names and email addresses. The demographic data collected at a later time was voluntary, and CHW/Ps were able to select “prefer not to answer” to every question. Champions in California assisted some participants by creating email accounts and completing the registration form, so they could participate in the training and evaluation. After completing the training, CHW/Ps were sent a web link to complete a 5–10-minute Qualtrics survey and received $50 compensation following survey completion. We were unable to provide hourly payment to offset time spent in the training.

### Data analysis

Descriptive analyses were performed to characterize participants in total and the subgroups of those who completed the English or Spanish course. Descriptive analyses were also completed on course characteristics. Similar to our evaluation of the independent online course [5], we used summary statistics to examine self-rated abilities and perceptions of the training. We performed two linear regressions, one with self-rated abilities as the outcome and the other with perceptions as the outcome to examine factors independently associated with survey ratings. Factors examined included demographics, course characteristics, the site where CHW/P participants were recruited from (Michigan, Florida, or California), and the Champion who facilitated the training. Along with exploring differences across preferred language, sites, and Champions, we conducted comparisons across racial/ethnic groups to determine significant differences among learners from different cultures as a proxy evaluation of cultural appropriateness.

## Results

### Training delivery characteristics

Trainings took place between March and October 2023. A successful training consisted of facilitating all five modules. Trainings could be delivered in a single day or across 2 days regardless of being virtual or in-person. The California site delivered 80% of all trainings in Spanish (*n* = 24; 19 Spanish; 5 English), whereas Florida led nearly all in English trainings (*n* = 31; 30 English; 1 Spanish), and Michigan ran only English trainings (*n* = 48). A total of 103 trainings were conducted, comprised of 26 single-day trainings, 38 two-day trainings, and one brief makeup session for a couple of learner. This resulted in 64 complete trainings across the 3 sites, 59 virtually (13 California, 22 Florida, and 24 Michigan) and 5 in-person (3 California, 1 Florida, and 1 Michigan). The number of trainings delivered by site differed because our primary goal was to deliver the training to at least 405 CHW/Ps across all sites rather than conducting a specific number of trainings at each site. The number of course takers varied for each training day, resulting in differences in the number of trainings conducted at each site to achieve the goal (see Table 2).

**Table 2.** Course characteristics by language version of the course

Characteristic	Total participants (*n* = 394)	Participants who took English course (*n* = 275)	Participants who took Spanish course (*n* = 119)	*P* value
Number of trainings by study site				<0.001
California	138 (35.0%)	21 (7.6%)	117 (98.3%)	
Florida	114 (28.9%)	112 (40.7%)	2 (1.7%)	
Michigan	142 (36.0%)	142 (51.6%)	0 (0%)	
Number of trainings completed				<0.001
Taken in 1 day	171 (43.4%)	102 (37.1%)	69 (58.0%)	
Taken over 2 days	222 (56.3%)	173 (62.9%)	49 (41.2%)	
Missing	1 (0.3%)	0 (0%)	1 (0.8%)	
Training modality				<0.001
Virtual	369 (93.7%)	265 (96.3%)	104 (87.4%)	
In-person	25 (6.3%)	10 (3.6%)	15 (12.6%)	

Trainings were conducted by 11 Champions and one Master Trainer who served as an emergency backup. While there was some variability in frequency and number of trainings across sites to complete the course, most Champions conducted at least one training per month for 2–5 months; eight Champions conducted between 3 and 12 trainings, while three Champions conducted only one training. The total time taken for the training, whether a single-day or 2 days, was a mean of 3.9 hours ± 1.1 hours (range 2–5.6 hours). Conducting one single training was shorter than a 2-day training, despite delivering the same material. The single-day mean was 3.5 hours ± 1.3. (range 2–5.6) compared to a mean of 4.2 hours ± 0.7 (range 2–5.5) for the 2-day training. For the 2-day training, the mean length of the first day was 2.2 hours ± 0.4 (range 1.5–2.8) and 2.0 hours ± 0.5 (range 1–3) for the second day.

The number of learners in the trainings ranged from 2 to 13, with a mean of 6.2 learners ± 2.9. More people took the 2-day training (56% versus 43% for 1 day). However, more Spanish course takers took the course in 1 day compared to English course takers (58% versus 37%). Ninety-four percent of participants took the training virtually, yet there was a higher proportion of Spanish course takers for in-person training compared to English course takers (13% versus 4%).

### Training fidelity

A total of 38 trainings (37% of all trainings conducted; 14 first training days and 24 s training days) were evaluated for fidelity. Of these fidelity assessments, 37 were completed by 5 Master Trainers (i.e., 2 at California, 2 at Michigan, and 1 at Florida). As none of the Master Trainers from Michigan or Florida were fluent in Spanish, a bilingual team member from Michigan completed the fidelity rating for a Spanish training conducted in Florida following instruction. All Champions except one consistently received ratings of 9 or 10 out of 10, demonstrating high fidelity. Most ratings of 9 (88%) resulted from not having an optimal recommended class size for group interaction (i.e., more or less than 5–10 learners). The one Champion who was rated lower underwent remediation and ultimately opted not to continue.

### Participant CHW/P characteristic

**Figure 1.**
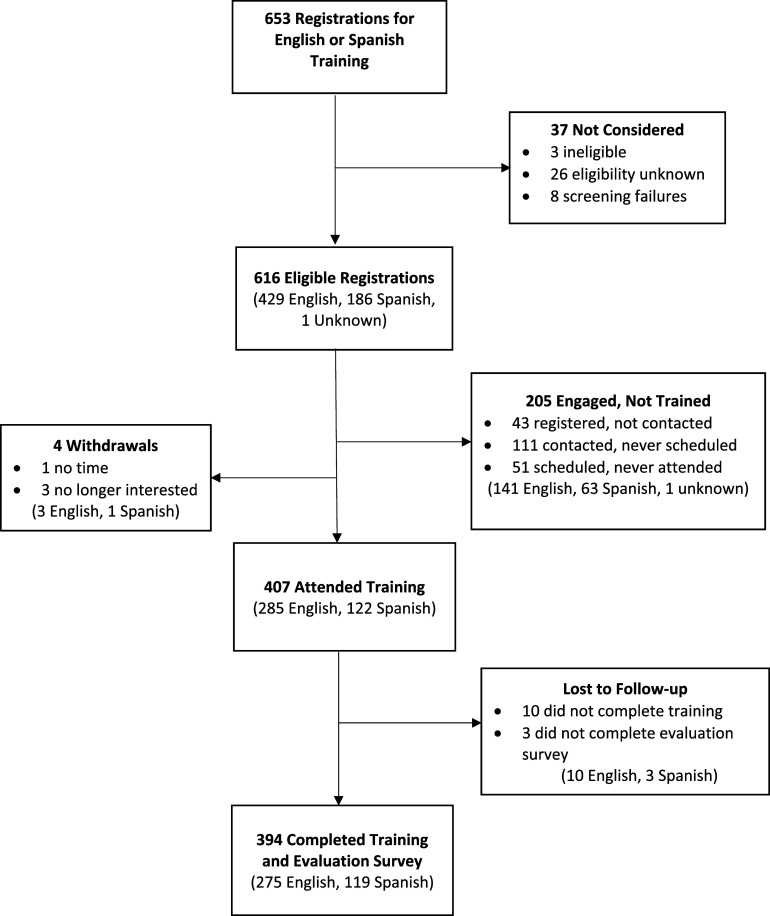
Participant flow chart for champion-led training and evaluation.

Figure 1 shows the flow of participants into the study. Participants in the California trainings were recruited and scheduled by the Champions from within their personal networks. Participants in Michigan and Florida were recruited primarily through contact with organizations in the state that represented or employed CHW/Ps and scheduled by site study team members. We received 653 registrations to complete the training course in either English or Spanish. Of these, 37 were not considered eligible for several reasons, including incomplete registration forms or not being a member of the target audience (i.e., a CHW/P). Of the 616 complete registrations, 407 completed at least some training, 4 withdrew, and 205 did not attend training. At the California site, 43 individuals registered but were not contacted because they were not recruited directly or able to be contacted by site Champions before the study training period was completed. By the study’s end, 394 participants completed the course and the evaluation survey.

Of the final sample of 394 participants, 142 were recruited from Michigan, 138 from California, and 114 from Florida. All sites primarily recruited participants from their statewide professional and personal networks, resulting in majority representation from those three states; however, recruitment was not limited to only those states. The Michigan and Florida sites relied more heavily on social media and word-of-mouth strategies, resulting in a broader reach than initially expected. For example, the Michigan site trained a few individuals from Idaho and Indiana, and Florida trainings included some participants from Georgia and Kentucky. Further, we did not specify that CHW/Ps needed to be currently employed by a community-based organization to participate. Therefore, CHW/Ps could belong to any number of community-based organizations, work independently, or be unemployed. The mean age of CHW/Ps was 41.6 years ± 13.8, and 80% reported female by gender and by sex on birth certificate. Slightly over half of the participants reported they were of Hispanic/Latino/Spanish origin, nearly all (98%) of whom were from the California site (See Table 3). Regarding race, 18% of the participants preferred not to answer or had missing data. Of the remainder of the participants, a plurality was White (40%) followed by Black (28%); approximately 14% reported another category – Asian, American Indian/Alaskan Native, some other race, or more than one race. Approximately 62% of the participants attained an Associate’s degree or higher, while 5% of the participants did not complete high school. Over half (52%) spoke English only, 32% were bilingual (i.e., English and Spanish), and 16% spoke Spanish only. Most were currently employed as a CHW/P (61%), 30% were volunteering as a CHW/P, and 22% reported being state-certified CHW/Ps. More than one-third of the participants (37%) reported having experience as a member of a research team that conducted community-based research.

**Table 3.** Participant characteristics (*N* = 394)

Characteristic	Total participants (*n* = 394)	Participants who took English course (*n* = 275)	Participants who took Spanish course (*n* = 119)	*P* value
Ethnicity				<0.001
Hispanic/Latino/Spanish origin	198 (50.3%)	89 (32.4%)	118 (99.2%)	
Prefer not to answer or missing	10 (2.5%)	9 (3.3%)	1 (0.8%)	
Race				<0.001
American Indian/Alaska Native	6 (1.5%)	6 (2.2%)	0 (0%)	
Asian	13 (3.3%)	13 (4.7%)	0 (0%)	
Black/African American	110 (27.9%)	110 (40.0%)	0 (0%)	
White	158 (40.1%)	102 (37.1%)	56 (47.1%)	
Some other race	28 (7.1%)	9 (3.3%)	19 (16.0%)	
More than one race	10 (2.5%)	7 (2.6%)	3 (2.5%)	
Prefer not to answer	69 (17.5%)	28 (10.2%)	41 (34.5%)	
Gender				0.02
Female	316 (80.2%)	210 (76.3%)	106 (89.8%)	
Male	70 (17.8%)	59 (21.5%)	11 (9.3%)	
Genderqueer, gender nonconforming, non–binary, neither exclusively male or female	3 (0.8%)	3 (1.1%)	0 (0%)	
Transgender	2 (0.5%)	1 (0.4%)	1 (0.8%)	
Prefer not to answer or missing	3 (0.8%)	2 (0.7%)	1 (0.8%)	
Sex on Birth Certificate (Female)	316 (80.2%)	213 (77.5%)	103 (86.6%)	0.04
Prefer not to answer or missing	9 (2.3%)	5 (0.02%)	4 (3.4%)	
Age, mean years (SD)	41.6 (13.8) (12 missing) 19–74	39.1 (13.7) (5 missing) 19–74	47.8 (11.9) (7 missing) 21–70	<0.001
Years working as a CHW/P, mean years (SD)	6.0 (5.9) (19 missing) 0–28	5.2 (5.6) (6 missing) 0–25	7.9 (6.3) (13 missing) 0–28	<0.001
Education				<0.001
< High school	21 (5.3%)	1 (0.4%)	20 (16.8%)	
High school diploma or GED	59 (15.0%)	17 (6.2%)	42 (35.3%)	
Some college	70 (17.8%)	57 (20.7%)	13 (10.9%)	
Associate degree	41 (10.4%)	36 (13.1%)	5 (4.2%)	
Bachelor’s degree	141 (35.8%)	111 (40.4%)	30 (25.2%)	
Masters or Doctorate	61 (15.5%)	52 (18.9%)	9 (7.6%)	
Missing	1 (0.3%)	1 (0.4%)	0 (0%)	
Languages spoken by CHW/Ps				<0.001
English only	203 (51.5%)	203 (73.8%)	0 (0%)	
Spanish only	64 (16.2%)	4 (1.5%)	60 (50.4%)	
Bilingual (English and Spanish)	126 (32.0%)	67 (24.4%)	59 (49.6%)	
Other	1 (0.3%)	1 (0.4%)	0 (0%)	
Professional status, n (%)				
Currently employed as a CHW/P	240 (60.9%)	179 (65.1%)	61 (51.3%)	0.03
State-certified as a CHW/P	86 (21.8%)	76 (27.6%)	10 (8.4%)	<0.001
Currently volunteer as a CHW/P	118 (29.9%)	61 (22.2%)	57 (47.9%)	<0.001
Experience as member of a research team conducting community–based research (Yes)	147 (37.3%)	111 (40.4%)	36 (30.3%)	<0.06

CHW/Ps = community health workers and promotoras; GED = general education development (i.e., high school equivalency diploma).

Almost all demographics differed by course taken. Compared to English course takers, Spanish course takers had a higher number of participants from Hispanic/Latino/Spanish origin (99% Spanish versus 33% English course takers) and who reported being some other race. Spanish course takers also had a higher proportion of females by gender (90% compared to 76%) and by sex on the birth certificate (87% compared to 78%), were older (mean age of 48 years compared to 39 years), and had fewer people attaining an Associate’s degree or higher (37% compared to 72%). In addition, half of the Spanish course takers were bilingual, compared to roughly one-quarter of English course takers (50% versus 24%). More Spanish course takers volunteered as CHW/Ps compared to English course takers (48% versus 22%); English course takers more often reported being currently employed as CHW/Ps compared to Spanish course takers (65% versus 51%). Fewer Spanish course takers were state-certified as a CHW/P compared to English course takers (8% versus 28%).

### Participant self-rated abilities and perceptions

**Figure 2.**
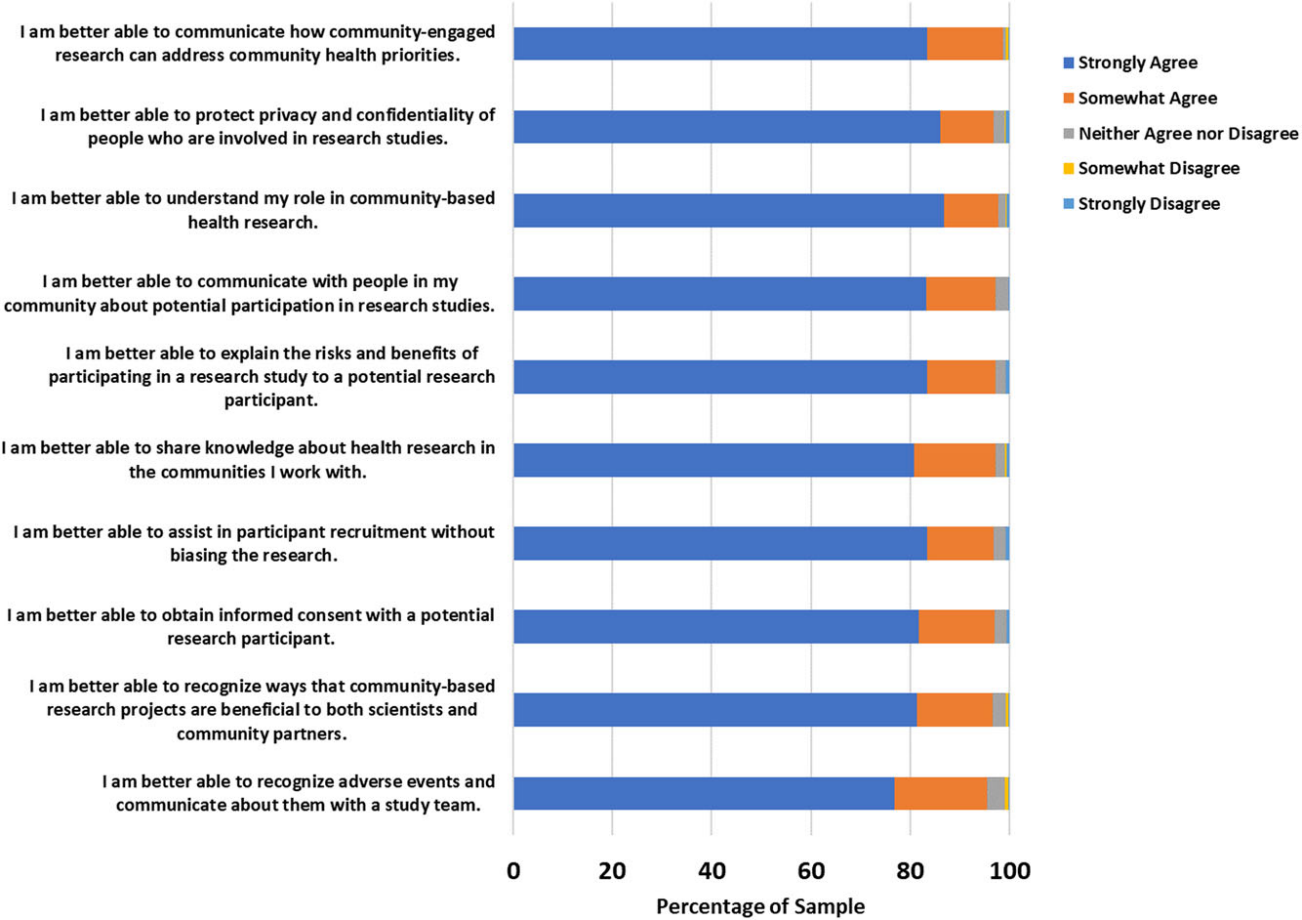
Self-rated abilities of CHW/Ps after facilitated training (*N* = 394).

**Figure 3.**
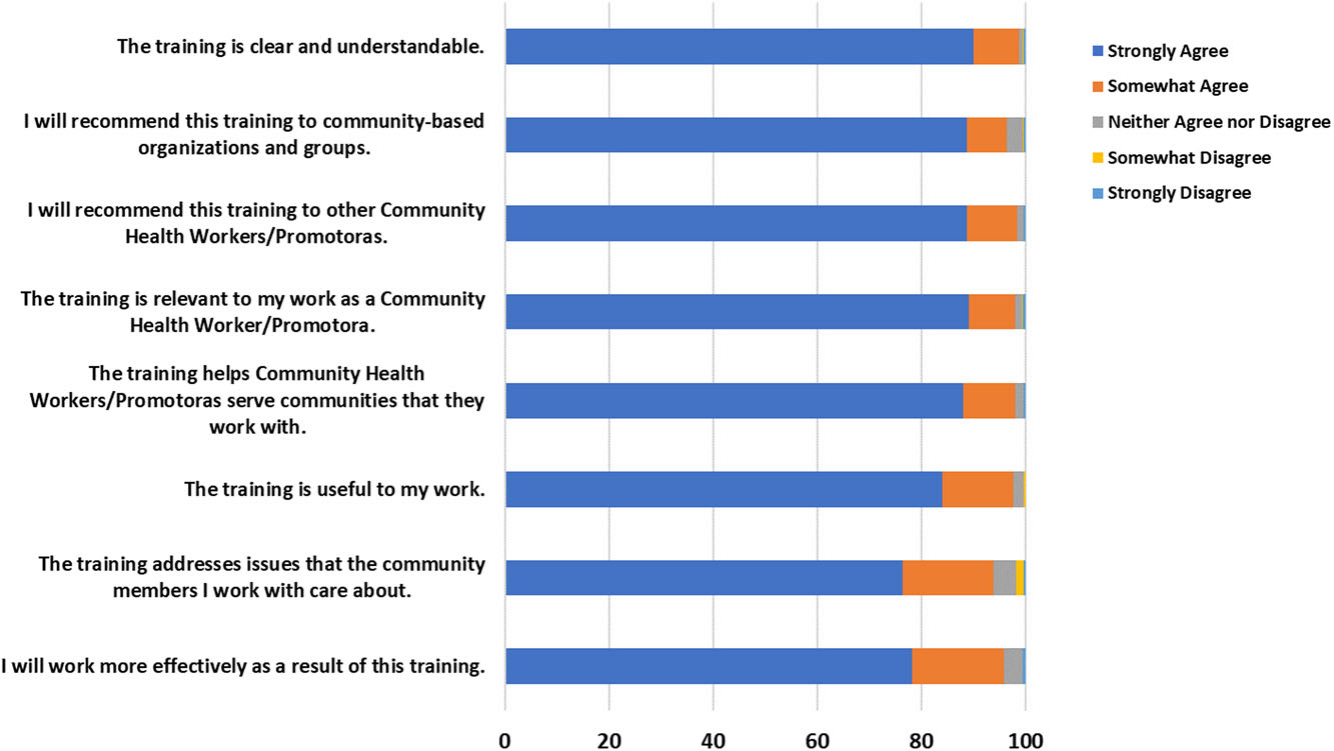
Community health workers/promotoras perceptions of the facilitated course (n = 394).

Between 95 and 100% of respondents reported improvement (strongly or somewhat agreed) in self-rated abilities (i.e., knowledge and skills for participating in research-related work) after the training (see Figure 2). Similar to our previous course evaluation ratings from the independent course participants [5], the most strongly agreed and somewhat agreed upon item was the ability to communicate how community-engaged research can address community health priorities, and the least strongly agreed and somewhat agreed upon item was the ability to recognize adverse events and communicate about them with the study team. Participants had positive overall perceptions of the course (see Figure 3). Nearly all CHW/Ps agreed that the course was relevant and felt the training was useful to their work (98% for both). Examining these scores as means (with a rating from 1 to 5 on agreement), the participants rated their abilities and perceptions very highly (4.8 ± 0.4) and (4.8 ± 0.3), respectively. Abilities were rated slightly better for Spanish course takers compared to English course takers (4.9 ± 0.4 versus 4.8 ± 0.5), but both are skewed highly to the strong agreement rating. Perceptions were not significantly different between English and Spanish course takers (4.8 out of 5 for both).

### Differences in outcomes by participant and site characteristics

We examined differences in ratings based on several factors in linear regression models. For the model with self-rated abilities as the outcome variable, only site was significantly independently associated with the outcome. California and Florida sites were rated both slightly higher than Michigan (*r* ^2^ = 0.04, *F* (2) = 7.21, *p* = 0.008). Within the California site, there was no difference between learners by ethnicity, but those who reported they were Hispanic/Latino/Spanish tended to have slightly higher scores compared to those who did not report being this ethnicity (*r* ^2^ = 0.03, *F* (1) = 3.87, *p* = 0.051). For the model examining perceptions of training, the only significant factor was site, with learners from Florida rating slightly higher than those in Michigan (*r* ^2^ = 0.03, *F* (2) = 4.90, *p* = 0.0079).

## Discussion

Our diverse multisite team developed and evaluated a culturally and linguistically tailored, peer-led research best practices course to provide a standardized training for the English- and Spanish-speaking CHW/P workforce. In this current study in which we evaluated the facilitated group training version, participants rated the training very favorably in terms of relevance and usefulness and reported an improvement in their self-rated abilities. These findings were similar to the independently taken online course version that consisted of the same content [5], providing support for either mode of delivery to address research training with the CHW/P workforce. Nonetheless, we found slight differences in outcomes of the facilitated training based on the training site, which warrants further discussion.

When researchers identify statistically significant differences in outcomes, it is critical to consider whether the differences are *meaningful* within the context of the study [8,9]. While we did identify two statistically significant differences among sites, the r^2^ values were small, and numeric differences in ratings ranged from 0.1 to 0.3 on a 5-point Likert scale, which we consider to be uninformative in terms of meaningfulness. Receiving a 4.6 or a 4.8 versus a 4.9 rating for course evaluations does not indicate contextually meaningful differences in participants’ self-rated abilities or perceptions of the course based on the primary language of the participant or the site leading the training. However, it is possible that the halo effect [10] may be responsible for the statistically significant finding. With the halo effect, a participant’s perception of the Champion facilitator may have influenced their rating of their own abilities or the course itself. Moreover, research has demonstrated that people are more comfortable and trusting of things with which they are familiar or that are provided by people with whom they are familiar [11]. Most Spanish-speaking participants at the California site were recruited directly from the personal networks of the Champions, which may have contributed to the higher ratings by those participants.

This research best practices course for CHW/Ps may be an important tool for community-engaged research teams because it fills gaps in CHW/P training expressed by researchers, research staff, and CHW/Ps themselves. For instance, in a study where research investigators who engaged CHW/Ps to be part of research teams were asked to prioritize research competencies that CHW/Ps need to know, the highest-rated competencies were related to implementation of research studies, such as privacy, confidentiality of data, understanding and adhering to a protocol related to random assignment of participants, and understanding the informed consent process [1]; these are all topics addressed by our research best practices course. Further, the ability to adequately collect and manage data has been mentioned as a training need by both researchers [4] and CHW/Ps [12]. This research best practices course could potentially reduce issues affecting research rigor when CHW/Ps are part of community study teams. In this study, our program evaluation was focused on self-ratings of abilities and perceptions of CHW/P learners. Future studies could be designed to examine process outcomes of research studies that involve CHW/Ps who have been trained using this course. Research process outcomes, measured through audits of data quality or assessment of adherence to study operations such as data confidentiality practices, could be important indicators of study rigor that may reflect research competencies by CHW/Ps working on study teams.

### Strengths & limitations

Regardless of the language of the training or whether it was conducted virtually or in-person, the training was rated highly, and CHW/Ps felt they had improved abilities related to research post training. The findings suggest that this training is culturally and linguistically appropriate for the intended group of CHW/Ps. Although we are unable to disentangle cultural and linguistic appropriateness in our evaluation, the course was robustly developed by a culturally and linguistically diverse team, our images and scenarios were chosen and created based on issues most prevalent in underserved communities (e.g., trust), and the overwhelming positive feedback from our culturally diverse sample, including Spanish speakers from communities across the country, contributes to our understanding of the appropriateness of the training. The course and all facilitated training materials resulted from a highly participatory process of development and refinement. In addition, particular attention was given to the Spanish translation and back translation into English by our team to ensure the use of appropriate phrases and tone [13,14]. Another strength was the evaluation of the training in both English and Spanish by a relatively large sample of CHW/Ps (*n* = 394) from different parts of the USA. The CHW/P Spanish speakers were mainly from the California site, which was due to difficulty in securing or retaining Spanish-speaking Champions at the Florida and Michigan sites. The majority of Spanish speakers from the California sample reflects the population in North-Central California and agency partners of the team at UC-Davis. One limitation is that we did not purposefully recruit participants actively employed by community-based organizations. In addition, we did not capture the names of organizations which employed the trainees. This resulted in a limited understanding of the organizations represented in our training cohorts and potential maximum reach of our training. Nonetheless, our sample aligned well with the demographics of CHW/Ps across the country. Another potential limitation of the study is the ceiling effect of positive ratings, which limits our ability to unpack variation in responses [10,15]. Moreover, we did not include a formal outcome measure examining Champion’s group process skills directly, so our conclusions about their role in adding to the effects of a group-based training are limited. However, we are further exploring this component in a qualitative evaluation that will be published separately. We also were unable to include any longitudinal measures of how the training impacted CHW/Ps’ ability to do their work, but this will be included in our future research on this topic. The facilitated training and all the training materials are planned to be disseminated on the website our team is developing and freely accessible. Because the facilitated training was designed to be executed using the community–academic partnership model described in this paper, it is unclear whether similar outcomes would be found if the training were implemented without oversight and training of Champions.

### Future directions

Qualitative and mixed methods analyses are currently underway to further examine the feedback we received and to identify opportunities for enhancing the training materials. Once these improvements are integrated, the facilitated training will be further piloted with sites that have community–academic partnerships, in different states than the original three study sites, to determine feasibility, facilitators, and barriers of conducting this training with CHW/Ps. Future efforts may focus on a larger implementation study to develop strategies to sustain this training over time. In addition, our team provided certificates of completion, but no knowledge assessment. During course development, our stakeholders mentioned that testing was not something they wanted for this training because it may discourage participation. Although not included currently in our course, another study of CHW/Ps utilized a knowledge assessment in a telehealth program, and all participants scored at least a 70% on the exam [16], supporting the idea that these assessments could be feasible for the CHW/P workforce.

## Conclusion

By engaging a diverse multisite team in an iterative refinement process, we developed a culturally and linguistically tailored research best practices training for CHW/Ps in English and Spanish. This standardized training, which was well received by CHW/P participants, can help to overcome the need for more consistent preparation of the CHW/P workforce to be a part of community-engaged research teams. The training and all materials will be broadly disseminated for use in the CTSA consortium and beyond.
